# Evaluation of mood disorder questionnaire positivity and associated factors in a population-based screening study

**DOI:** 10.1186/s41155-022-00229-9

**Published:** 2022-08-11

**Authors:** Numan Konuk, Elif Karaahmet, Ülkem Angın, Alperen Kılıç, Zekeriya Kökrek

**Affiliations:** 1 Department of Psychiatry, Faculty of Medicine, Düzce University, Yörükler Mah., Konuralp Campus, Merkez, Düzce, 81620 Istanbul, Turkey; 2 Department of Psychology, Faculty of Science and Literature, Haliç University, Sütlüce Mah., İmrahor Cd., No. 82, Sütlüce, 34445 Istanbul, Turkey; 3 Prof. Dr. Cemil Taşçıoğlu City Hospital, Department of Psychiatry, Ministry of Health Istanbul Provincial Health Directorate, Kaptan Paşa, SSK Okmeydanı Hst., No. 25, Şişli, 34384 Istanbul, Turkey; 4 Department of Psychiatry, Faculty of Medicine, Istanbul Medipol University, TEM Avrupa Otoyolu Göztepe Çıkışı No: 1, Bağcılar, 34214 İstanbul, Turkey; 5 Department of Psychology, Faculty of Humanities and Social Sciences, İstanbul Ticaret University, Sütlüce, İmrahor Cd. No. 90, Beyoğlu, 34445 Istanbul, Turkey

**Keywords:** Bipolar disorders, Epidemiology, Mood disorders questionnaire, Population-based study, Turkish people

## Abstract

The Mood Disorders Questionnaire (MDQ) is a 3-item scale that is frequently used in bipolar disorders (BD) screening and questions the symptoms of BD, its effect on functionality, and the coexistence of symptoms. The aim of this study is to evaluate the prevalence of positive screening of the MDQ among general population and to investigate the associated risk factors.

In this cross-sectional study, the sample was randomly selected from household data to represent the city population. A total of 432 participants were asked to fill in MDQ, CAGE (cutting down, annoyance by criticism, guilty feeling, and eye-openers) questionnaire, which consists of four clinical interview questions proven to aid in the diagnosis of alcoholism, and clinical and sociodemographic data form.

The Cronbach’s alpha value of our current study was 0.813 for MDQ. The prevalence of MDQ positivity was found 7.6%. The estimated prevalence rate of bipolar disorders varied between 0.3 and 13.4% according to different cut-off values. Multivariate logistic regression models showed that the presence of possible alcohol addiction, shift work history, and body mass index (BMI) were statistically significant predictors of MDQ positivity.

The prevalence of MDQ positivity found is similar to studies in literature. Keeping in mind that psychometric properties of the MDQ, positive screen results should be cautiously interpreted due to the presence of other risk factors and comorbidities.

## Introduction

Bipolar disorders (BD) are frequent, serious mental illnesses associated with considerable morbidity and mortality (Frye et al., 2005). Apart from the Diagnostic and Statistical Manual of Mental Disorders (DSM-IV; American Psychiatric Association, 2000; First, 1997) diagnostic features of Bipolar I (mania and depression) and Bipolar II (hypomania and depression), the concept of bipolar spectrum disorders (BSD) comprises of a range of bipolar conditions with less evident manifestations. Furthermore, the estimated lifetime prevalence rates of BD range between 3% and 6.5% community based in screening studies (Kessler et al., 1994).

However, the prevalence of bipolar disorders in Turkey has not been explicitly investigated. It is important to estimate the prevalence of BD correctly since underdiagnosis or overdiagnosis of the disorder would likely lead to negative consequences such as increased morbidity and mortality as well as higher socioeconomic costs (Zimmerman et al., 2011). The Structured Clinical Interview for DSM-IV (SCID) has been the gold standard in the correct diagnosis of BD, but it is not practical to use in population settings. On the other hand, screening instruments have been criticized for being used as “case finder instruments” rather than screening instruments (Zimmerman et al., 2011; Hirschfeld et al., 2000). Inappropriate use of Mood Disorders Questionnaire (MDQ) could mistakenly result in a much higher prevalence of BD than the real prevalence in a population (Zimmerman et al., 2011). A recent population-based study in the UK found a lifetime prevalence of 1.7% in MDQ screening. (Humpston et al., 2021). There are studies in the literature criticizing inappropriate conclusions regarding the prevalence, morbidity, and under-recognition of BD drawn from the use of MDQ as a diagnostic proxy. It means a positive screening test indicates the possibility that the individual in question may have the suspected disease or condition, which can only be confirmed by a diagnostic test (Bowden et al., 2007; Zimmerman et al., 2004; Zimmerman & Galione, 2011; Ketter, 2010).

It has been stated in the literature that there is a reciprocal relationship between the clinical and physiopathology of BP and shift work, alcohol and substance use, and obesity (Cole et al., 1990; Kessler, 1995; Calkin et al., 2009).

Shift work is suggested to increase the risk of developing or aggravating mood disorders, especially in vulnerable individuals (Cole et al., 1990). Kessler et al. (1994) have reported that 12 months prevalence of bipolar disorders for workers was 1.1%, while the prevalence of depression was 6.4%. Although the prevalence of bipolar disorders was much lower, the lost workdays were strikingly higher.

Epidemiological data have shown that alcohol or substance use disorders are 5–6 times more likely to have a history of bipolar disorders than subjects without substance use disorders (Kessler, 1995; Kessler et al., 1997).

Finally, several studies have reported a higher prevalence of obesity in patients who have a bipolar disorder. Although the causal effect of this association remains undetermined, findings suggest that body mass index (BMI) is associated with the prognosis and the outcome of bipolar disorders (Calkin et al., 2009).

As currently, there is no epidemiological study in the literature based on country population that has investigated the prevalence of BD in Turkey; we conducted this study on a representative sample of the population of Zonguldak, a midsize coal miner city in Turkey. This article also discusses the psychometric properties of the MDQ as well as the prevalence of bipolar disorders and associated risk factors such as shift workers, alcohol addiction, BMI, and another factors.

## Methods

### Sampling

Zonguldak is a special mining city where a significant part of the population works in shifts. This cross-sectional study was conducted in the city of Zonguldak, which has a population of 107,354, according to the population census in the yearbook (TÜİK, 2007). According to the average household size of 4.1 people in the city, the number of houses was calculated as 26,184. It was calculated that the sample should include 534 houses based on 15% expected MDQ (+) with a 3% sample error and 95% confidence interval. 586 participants aged between 15–75 in 534 houses were selected by stratified random sampling from household data to represent the city population. While choosing the registered house numbers in the headman's office, simple random sampling was done by using the table of random numbers. The study participants were visited without informing them in advance after confirming the addresses with local health clinic staff and local authorities. All residents of the appropriate age range in each household were determined, and those who were present were invited to participate in the study. For the individuals who could not be reached in two visits within the same day, random addresses in the same section of the city were picked from the previously determined backup list. The backup participants were chosen from the same gender of the original randomly selected individual. The surveys were carried out by two research assistants and intern physicians, who were trained on the investigation tools used in the study.

All of the participants voluntarily signed written and informed consent to participate in the study. A total of 432 participants were given and asked to fill in sociodemographic data form, CAGE, and MDQ scale.

One hundred fifty-four people who were not included in the study were evaluated as random missing. Among those not included in the analysis, 19 people were under the age of 15 or over the age of 75, 35 were illiterate, 10 filled out the data incompletely, and the remaining refused to participate in the study. There was no significant difference in terms of sociodemographic variables between 154 people who were not included in the analysis and those who were included in the analysis.

### Instruments

#### Sociodemographic data form

We used a data form, developed by the authors, that included questions about age, gender, height, weight, socioeconomic status, smoking habits, and work shift schedule.

#### Mood Disorder Questionnaire (MDQ)

This questionnaire was developed by Hirschfeld et al. (2000). They found that individual item correlations with total score on the MDQ range from 0.50 to 0.75 (Cronbach alpha coefficient: 0.90). Also, the Cronbach’s alpha value of our current study was 0.813 for MDQ. The Turkish adaptation and standardization of the MDQ was done by Konuk et al. (2007) as part of this study. According to this research, the ideal cut-off score of the scale was found to be 7 (the cut of point 7 had 0.64 sensitivity and 0.77 specificity, the cut-off point 5 had 0.81 sensitivity and 0.53 specificity, and the cut-off 6 had 0.75 sensitivity and 0.63 specificity). It is based on DSM-IV (American Psychiatric Association, 2000; First, 1997) and consists of 3 “yes and no” questions answered by the subject. The first question includes 13 subquestions and examines the lifelong history of manic and hypomanic symptoms, besides effect, irritability, sleep, libido, thinking, attention, energy level, and behavior problems. The second question asks whether the symptoms yielded “yes” answers in the first section occurred during the same period or not. The third question explores if the symptoms impacted significantly on the daily functioning of the individual. Hirschfeld et al. (2000) used the 5-item version of the MDQ scale in their study, and we included these two questions in the analysis to test the effects of item 4 (positive family history of BD ) and item 5 (whether previously diagnosed with BD or not) questions on the estimated prevalence. The role of these two questions in getting direct positive answers in screenings are discussed in the literature (Zimmerman et al., 2011; Hirschfeld et al., 2000).

#### CAGE

It is a screening instrument that is well suited for use in busy medical settings where there is limited time for patient interviews. It uses 4 straight forward yes/no questions that clinicians can easily remember. CAGE can be self-administered or conducted by a clinician, and its utility is proven for use in routine health screening of adults and adolescents over the age of 16. The screen may identify individuals with alcohol problems that may have been otherwise missed. Item responses on the CAGE are scored as 0 or 1, and a total score of 2 or greater is considered clinically significant for a potential alcohol addiction issue (Ewing, 1984; Arıkan et al., 1991).

### Statistical analysis

All numerical data were expressed as mean ± standard deviation, and descriptive analysis was used to express all categorical variables as numbers and percentages (*n*, percent). According to descriptive statistics, sociodemographic characteristics of the 432 subjects who participated in the survey, 201 were male (46.5%), and 231 (53.5%) were female. 14 (3.5%) participants were illiterate, and thus their forms were filled by guidance from the researchers. One hundred twenty-one (30.1%) participants were elementary school graduates. The number and proportion of the education level of participants (elementary, secondary, high school, and college), socioeconomic status, and marital status are displayed in Table 1. More than half (59.2%) of the participants were in the middle class, and 87.5% lived in an urban area. The mean age of the study group was 36.82 (SD 13.75), and the median was 36 (min. 16, max. 70).

**Table 1:** Sociodemographic characteristics of the study population

Variable	Total (***n*** = 432) ***n*** (%)
**Gender**	
Male	201 (46.5)
Female	231 (53.5)
**Education**	
Illiterate only	14 (3.5)
Elementary school	121 (30.1)
Secondary school	71 (17.7)
High school	130 (32.33)
College educated	66 (16.4)
**Household income**	
High	147 (15.3)
Middle	568 (59.2)
Low	247 (25.5)
**Setting**	
Urban	344 (87.5)
Rural	49 (12.5)
**Marital status**	
Single	119 (29.2)
Married	265 (65)
Separated or divorce	11( 2.7)
Widow/widower	13 (3.2)

Firstly, univariate logistic regression analysis was performed to identify the specific contributions of appropriate independent variables (age, gender, marital status, educational status, setting, alcohol addiction, smoking, shift work, and BMI) to our dependent variable (MDQ positivity). Then, the factors that were significant in this analysis (age, gender, marital status, alcohol addiction, smoking, and shift work) also BMI, were included in the multivariate logistic regression analysis on the dependent variable.

Expected prevalence percentage for several cutoff score was calculated according to sensitivity values reported in previous study (Konuk et al., 2007). All the tests were two-tailed with 5% level of significance. Data were analyzed using SPSS Version 22 (SPSS V.22.0). All procedures followed were in accordance with the ethical standards of the responsible committee on human experimentation (Research Ethics Committee of University approved the study protocol) and with the Helsinki Declaration of 1975, as revised in 2000.

## Results

The Cronbach's alpha value of our current study was 0.813 for MDQ. The expected prevalence of MD positivity varied between 10.3 to 13.4% according to different cutoff scores in item 1 and positive response to item 2 and item 3 (Table 2) Furthermore, when item 4 and item 5 were added to the first 3 questions positivity, prevalence rates were decreased (Table 3).

**Table 2:** The expected prevalence of MDQ positivity according to different cut off value of item 1 and positive response to items 2 and 3

Cut-off	Frequency	Percent (%)^a^	Expected prevalence %^b^
5	46	10.6	13.1
6	39	9.0	12.0
7	33	7.6	11.9
8	27	6.3	13.4
9	16	3.7	10.3

^a^The frequency according to different cut-off scores were found from positive responses to item 1 + item 2 + item 3

^b^Expected prevalence % was calculated according to sensitivity values reported in (Konuk et al., 2007)

**Table 3:** The expected prevalence of MDQ positivity according to items 4 and 5 when adding to the first three item positive response at different cut off value

Cut-off^a^	Item-4 (+) Expected prevalence^b^	Item-5 (+) Expected prevalence^b^	Both Item 4 and 5 (+) Expected prevalence^b^
5	2.0	3.2	0.9
6	1.6	2.6	0.3
7	1.9	3.0	0.4
8	1.5	1.5	0.5
9	1.9	1.9	0.6

^a^The frequency according to different cut-off scores were found from positive responses to first three items of MDQ plus item 4, item 5, and both item 4 and item 5. ^b^Expected prevalence % was calculated according to sensitivity values reported in (Konuk et al., 2007)

In univariate logistic regression analysis, being female, married, and increasing age were statistically significantly decreasing the probability of MDQ positivity. On the other hand divorced, alcohol addiction, smoking, and shift work history were statistically significantly increasing the MDQ positivity. In univariate analysis, educational status (OR = 1.40; 95% Cl = .45, 4.43; *p* = .53), setting (OR = 1.00; 95% Cl = .33, 2.98; *p* = 1.00), and BMI (OR = .925; 95% CI = .85, 1.00, *p* = .06) were not found to be statistically significantly when associated with MDQ positivity (Table 4). Although BMI was not statistically significant, it was included in the multivariate logistic regression model.

**Table 4:** Univariate logistic regression analysis

	OR	95% C.I. for OR
**Age**	.964*	.935–.993
**Gender**		
Male	1.00	
Female	.474*	.227–.989
**Marital status**		
Single	1.00	
Married	.384*	.183–.806
Widow	.000	
Divorced	1.417*	.280–7.161
**Education status**		
Elementary	.938	.101–8.718
Secondary	.530	.148–1.904
High	1.334	.402–4.430
College	1.485	.511–4.318
**Setting**		
Urban	1.00	
Rural	1.00	.335–2.984
**Alcohol addiction**		
No	1.00	
Yes	4.304*	1.645–11.264
**Cigarette use**		
No	1.00	
Yes	3.669*	1.726–7.798
**Shift-worker**		
No	1.00	
Yes	3.614*	1.357–9.625
**BMI**	.925	.853–1.005

When positive responses are given to item 1 + item 2 + item 3

*BMI* body mass index, *OR* odds ratio, *CI* confidence interval

*P value less than .05

After adjusting for other variables in the multivariate logistic regression model, only BMI, shift work history, and alcohol addiction were found to be independent predictors of MDQ positivity (Table 5). It was observed that alcohol addiction and shift work history statistically significantly increased MDQ positivity. Positive MDQ participants were 6.67 times more likely to have a shift work and 3.38 times more likely to alcohol addiction compared to negative MDQ cases. One unit increase in BMI was associated with a 0.85 times decrease in MDQ positivity probability.

**Table 5:** The factors associated with MDQ positivity in multivariate logistic regression analysis

	***P*** value	OR	95% C.I. for OR	
			Lower	Upper
**BMI**	.005*	.847	.755	.951
**Shift worker**	.006*	6.670	1.722	25.837
**Alcohol addiction**	.028*	3.384	1.141	10.038

When positive responses are given to item 1 + item 2 + item 3

*BMI* body mass index, *OR* odds ratio, *CI* confidence interval

*P value less than .05

## Discussion

Epidemiological prevalence studies of BD have been carried out in most major populations around the globe, yet to—our knowledge—this is the first study in the literature investigating the prevalence of BD in a representative Turkish population. The prevalence rates in the diagnosis of bipolar disorder may vary due to cultural differences, although our findings show similar rates with a recent study (Humpston et al., 2021). It has been reported in the literature that prevalence of bipolar disorders is around 1%.

MDQ was developed by Hirschfeld et al. (2000), and the Turkish version was studied by Konuk et al. (2007) on psychiatry outpatients. On the other hand, rightfully, Zimmerman has written that the MDQ has been used as “a case finder instrument” rather than a screening test (Zimmerman et al., 2011). This was because some researchers presented MDQ positivity as a formal BD diagnosis and argued that high MDQ positivity demonstrates higher BD rates (Das et al., 2005). Therefore, it is necessary to consider positive and negative predictive values as well as sensitivity and specificity for any screening tool.

Our results were interpreted based on our previous work sensitivity (0.64) and specificity (0.77) values and Hirschfeld et al. (2000) first validation study criteria for cut-off point seven, which included: seven positive responses in item 1, positive item 2 (symptoms occurring together), and moderate to a high degree of functional impairment in item 3. According to the criteria of the Turkish validation study (Konuk et al., 2007) for cutoff point seven, the expected prevalence in the Turkish general population was 11.9%, which falls in the range of previous MDQ screening study results (Hirschfeld et al., 2000). There was no statistically significant difference between genders at all cutoff points (*p* > 0.05). When we include positive item 4 requirement (i.e., Do you have any family member with BD) to criteria, at seven cutoff point, the prevalence decreases to 1.9% with a sensitivity of 64% according to the previous study (Konuk et al., 2007). Requiring positive item 5 (personal history of BD diagnosis), but not positive item 4 response results in 3% and requiring both item 4 and item 5 to be positive besides the positive response to the first 3 questions, diminishes the prevalence to 0.4%.

MDQ was criticized for not being sensitive enough to detect bipolar II and other bipolar spectrum conditions, when Q3 functional impairment was limited to a severe and moderate degree (Yang et al., 2014). When all of the situations mentioned above are considered, this first BD epidemiological study in a Turkish population places BD prevalence rate at 0.3 to 13.4% range according to MDQ positivity. The sensitivity of MDQ to detect bipolar I condition is higher compared to bipolar II, and bipolar Not Otherwise Specified (NOS), and the last two diagnoses might likely be missed by MDQ screening, especially if item 3 answers include moderate and severe functional impairment and not a mild impairment. In this situation both item 3 and cutoff points in item 1 answers play a role in decreasing sensitivity. However, they also improve the specificity of the scale. When the cutoff point is decreased, or mild functional impairment is included, MDQ tends to identify more false positive cases with especially concurrent conditions highly associated with BD, such as borderline personality (Galione & Zimmerman, 2010; Parker et al., 2012; Zimmerman et al., 2010 ), anxiety (Parker et al., 2012; van den Berg et al., 2010), or substance abuse disorders (Zimmerman et al., 2011; Parker et al., 2012; van Zaane et al., 2012; Murray & Lopez, 1996) in literature or smoking habits, and shift worker conditions (Cole et al., 1990) as in the current study. Contrary to the literature (Calkin et al., 2009), in our study, BMI did not increase but decrease MDQ positivity, however, BMI values of individuals should be taken into account as it affects MDQ positivity.

The study was conducted in a mining city, where most of the population works on a shift schedule. To investigate potential confounding factors that might be influencing MDQ positivity (positive response to item 1, 2, and 3), we conducted regression analysis among the participants according to age, gender, marital status, education status, setting, alcohol addiction, cigarette use, shift work, and BMI. The participants with positive MDQ were found to have 3.38 and 6.67 times (OR) higher risk to alcohol addiction and work in a shift schedule, respectively, according to multivariate logistic regression analysis. According to the same criteria, one unit increase in BMI resulted in a 0.85 times drop in MDQ positivity.

A known problem in investigating the prevalence of BD by using MDQ is that the prevalence rates may differ depending on the study population, such as clinical versus the general population. A limitation of this study is that the Turkish version of MDQ was validated in individuals who presented to an outpatient psychiatric clinic rather than the general population. We also had to rely on only MDQ positivity for BD diagnosis, as we could not conduct a psychiatric evaluation of participants to confirm bipolar diagnoses. Our sample size was small, but it had strong representation, as we randomly chose the addresses from each section of the city according to the population of each section.

According to the optimal cutoff point 7 of MDQ, our study yielded a range of 0.4-11.9% prevalence rate for BD in the Turkish population. However, the findings of the present study should be considered based on the aforementioned limitations. When related factors such as shift work schedule, alcohol addiction, and BMI features of a selected sample, and presence of other uninvestigated confounding factors are considered in addition to the degree of functional impairment (changing prevalence of bipolar II and bipolar NOS cases) and other operating characteristics of MDQ; positive MDQ results may not only show BD, but possibly also other conditions. As a result, one should avoid interpreting our results to make statements about overdiagnosing or underdiagnosing BD based on MDQ positivity.

## Conclusions

According to the our first community-based Turkish study, the expected BD prevalence varies between 0.3 and 13.4. This variability can be explained by the psychiatric comorbidity or subsyndromal cases as well as due to BMI or other psychosocial factors (i.e. alcohol addiction and shift worker. Also, the inclusion of item 4 and item 5 of the MDQ results in changes in the psychometric properties of the scale. This should be taken into consideration when choosing to the purpose of the use of the MDQ. Therefore, when interpreting screening results with MDQ, imperfect detection and over-diagnosis should be interpreted together. More research is needed to determine the strengths and limitation of the psychometric properties of the MDQ to screen in a community-based research.

## Data Availability

The datasets used and/or analyzed during the current study are available from the corresponding author on reasonable request.

## Abbreviations

MDQ: Mood Disorders Questionnaire
BD: Bipolar disorders
BSD: Bipolar spectrum disorders
SCID: The Structured Clinical Interview for DSM-IV
BMI: Body mass index
NOS: Not otherwise specified
